# Design and Evaluation of a Percutaneous Fragment Manipulation Device for Minimally Invasive Fracture Surgery

**DOI:** 10.3389/frobt.2019.00103

**Published:** 2019-10-30

**Authors:** Ioannis Georgilas, Giulio Dagnino, Beatriz Alves Martins, Payam Tarassoli, Samir Morad, Konstantinos Georgilas, Paul Koehler, Roger Atkins, Sanja Dogramadzi

**Affiliations:** ^1^Department of Mechanical Engineering, University of Bath, Bath, United Kingdom; ^2^The Hamlyn Centre for Robotic Surgery, Imperial College London, London, United Kingdom; ^3^Instituto de Biofísica e Engenharia Biomédica, Faculdade de Ciências da Universidade de Lisboa, Lisbon, Portugal; ^4^University Hospitals Bristol NHS Foundation Trust, Bristol, United Kingdom; ^5^School of Life & Health Sciences, Aston University, Birmingham, United Kingdom; ^6^School of Engineering, University of Aberdeen, Aberdeen, United Kingdom; ^7^Bristol Robotics Laboratory, University of the West of England, Bristol, United Kingdom

**Keywords:** biomechanical testing, robot-assisted orthopedic surgery, fracture reduction, cadaveric trials, surgical tracking

## Abstract

Reduction of fractures in the minimally invasive (MI) manner can avoid risks associated with open fracture surgery. The MI approach requires specialized tools called percutaneous fragment manipulation devices (PFMD) to enable surgeons to safely grasp and manipulate fragments. PFMDs developed for long-bone manipulation are not suitable for intra-articular fractures where small bone fragments are involved. With this study, we offer a solution to potentially move the current fracture management practice closer to the use of a MI approach. We investigate the design and testing of a new PFMD design for manual as well as robot-assisted manipulation of small bone fragments. This new PFMD design is simulated using FEA in three loading scenarios (force/torque: 0 N/2.6 Nm, 75.7 N/3.5 N, 147 N/6.8 Nm) assessing structural properties, breaking points, and maximum bending deformations. The PFMD is tested in a laboratory setting on Sawbones models (0 N/2.6 Nm), and on *ex-vivo* swine samples (*F* = 80 N ± 8 N, *F* = 150 ± 15 N). A commercial optical tracking system was used for measuring PFMD deformations under external loading and the results were verified with an electromagnetic tracking system. The average error difference between the tracking systems was 0.5 mm, being within their accuracy limits. Final results from reduction maneuvers performed both manually and with the robot assistance are obtained from 7 human cadavers with reduction forces in the range of (*F* = 80 N ± 8 N, *F* = 150 ± 15 N, respectively). The results show that structurally, the system performs as predicted by the simulation results. The PFMD did not break during *ex-vivo* and cadaveric trials. Simulation, laboratory, and cadaveric tests produced similar results regarding the PFMD bending. Specifically, for forces applied perpendicularly to the axis of the PFMD of 80 N ± 8 N deformations of 2.8, 2.97, and 3.06 mm are measured on the PFMD, while forces of 150 ± 15 N produced deformations of 5.8, 4.44, and 5.19 mm. This study has demonstrated that the proposed PFMD undergoes predictable deformations under typical bone manipulation loads. Testing of the device on human cadavers proved that these deformations do not affect the anatomic reduction quality. The PFMD is, therefore, suitable to reliably achieve and maintain fracture reductions, and to, consequently, allow external fracture fixation.

## Introduction

The incidence of fractures of the lower limb, especially osteoporotic, is increasing and their surgical treatment accounts for a large proportion of orthopedic operations (Hernlund et al., 2013). These type of fractures bear considerable health costs and if managed sub-optimally could have a detrimental effect to patient's quality of life (Joubair et al., 2015). The standard fracture procedure can be summarized in two steps: (1) fracture reduction, and (2) fixation of bone fragments with a stable mechanical construct such as an intramedullary nail or using plates and screws. The reduction involves manipulating bone fragments to restore the anatomical bony alignment as precisely as possible. To ensure accurate reduction, the preferred practice is to use open surgery, which is, however, associated with extensive soft tissue damage, delayed fracture union, and increased risk of infection (Marsh, 2015). As an alternative, percutaneous techniques have been developed to mitigate these problems (Gaston et al., 2005).

Percutaneous techniques use a combination of pins, screws and wires (K-wires) inserted through the patient's skin into the fragments. The pins are used as “handles” to move the fragments to correct positions. The forces involved in fragment manipulation can be substantial (Harms et al., 1999), often not translating to the fragments but instead deforming (e.g., bend, twist, buckle) the inserted hardware or not creating the firm hardware-bone bond, thereby decreasing the required reduction accuracy. To address these issues different designs have been proposed for percutaneous fragment manipulation devices (PFMD).

Modern pin designs facilitate easy insertion of the external fixator pins in urgent situations and aim to maintain pin-bone construct stability through a combination of the immediate mechanical interface and subsequent bio-integration (Moroni et al., 2002). This is achieved by employing a self-tapping (and in some cases self-drilling) design (Green et al., 2010), with a coating which promotes osseo-integration; hydroxyapetite (HA) (Moroni et al., 1998). Self-drilling designs however are less preferable (up to 25%) when compared with pre-drilled pins (Andrianne et al., 1987).

Larger pin diameters naturally confer a high bending rigidity, however pins >6 mm in diameter may result in stress fractures through the pinhole and are therefore avoided (Capper et al., 1993). In drilled pin designs, the pilot hole will be placed with a slight mismatch with the greater thread diameter of the pin to increase torque resistance and pull-out strength by radially preloading the pin-bone interface. However, large mismatches of >0.4 mm may lead to micro fractures by exceeding the cortical bone's elastic limit (Biliouris et al., 1989). In hard cortical bones, pins (or screws) with a small pitch height and a low pitch angle are used, whereas in softer cancellous bone pins with threads of higher pitch vertex angles and larger thread diameters are employed (Chapman et al., 1996) to increase pullout strength.

External fixator arrangements are often used to stabilize the pins and ensure a secure grip to the bone fragment. Füchtmeier et al. (2004) proposed one such PFMD for use with the “RepoRobo” system consisting of two Seldrill Screws with mono-cortical insertion into the distal shaft fragment interconnected by a carbon fiber rod joined by means of an open tube-to-tube clamp. A customized 2-finger gripper (Schunk Co. Ltd; PGS 100) was used to secure this arrangement. Similarly, Gösling et al. (2006) used two Schanz Screws (5 mm diameter) connected via an external fixator tube. The tube was connected to an instrumented handle with a load cell to allow force measurement during manual manipulation. A similar configuration for a robotic application is proposed by Ye et al. (2012), but instead of a gripper the manipulation device is integrated with the robotic mechanism and two screws are inserted into the manipulated fragment.

An alternative to external fixator configurations is to use locking plates and screws (Cronier et al., 2010). Schmucki et al. (2004) described two versions of a reposition plate developed by the AO Development Institute (ADI, Davos, Switzerland), each utilizing two or three mono-cortical locking screws at oblique angles to the plate to ensure a stable connection with the fragment. In the same work, a third manipulation device is proposed that combines a central bi-cortical pin with three crossed Schanz screws (3 mm diameter) and a tension ring.

A combination of the external fixation and locking options proposed by Weber-Spickschen et al. (2010) is a *Three-Point-Device* composed of a frame that is distally attached with two mono-cortical screws, while a third Schanz screw was placed ventral to the fragment. In the reported results this approach outperforms all other solutions in terms of the attachment stability.

Although these solutions are plausible for the reduction of long bones, they are difficult to implement for reduction of intra-articular fractures where fragments have reduced bone volume making it difficult to insert a manipulation pin. Additionally, the insertion would have to be mono-cortical since there is only one cortical plane available in most small bone fragments. All these lead to the conclusion that a PFMD should have a reasonably smaller geometry, which, on the other hand, can lead to significant bending deformations under manipulation forces that can be extremely high (Georgilas et al., 2015). The potential deformations, consequently, can have a detrimental effect to reduction accuracy.

Within this context, the current work of this group is concerned with designing a new PFMD for intra-articular fragments that can sustain the manipulation forces. PFMD performance has been assessed under different loading and tested in simulation, on artificial bone models and in cadaveric models. Device design and different assessment results are presented here. To evaluate deformation characteristics of all parts (i.e., PFMD, fragment, femoral shaft), optical tracking methods were used, which were subsequently validated with electromagnetic tracking. The PFMD is primarily designed for use in a robot-assisted image-guided approach (Dagnino et al., 2017a), and the robotic mechanism is used for the loading scenarios in the *ex-vivo* and cadaveric model experiments. Furthermore, the same PFMD, with an attached handle, has also been used in the *ex-vivo* and cadaveric trials by an experienced surgeon to perform manual reductions of distal femur fractures, showing applicability of the device for manual fracture reductions as well.

## Methods

### Manipulation Device

**Figure 1:**
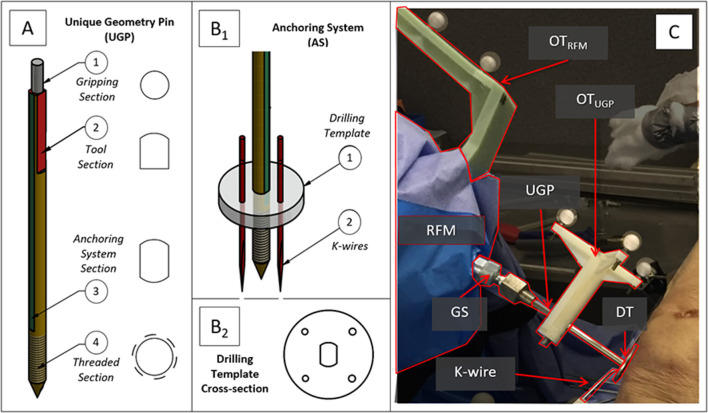
Robot-bone fixation system. **(A)** CAD drawings of the Unique Geometry Pin (UGP) and its different cross-sections, **(B**_**1**_**)** the anchoring system (AS), and **(B**_**2**_**)** a detail of the Drilling Template (DT). **(C)** The UGP is secured in the Gripping System (GS) and connects the RFM end-effector with the bone fragment. Optical tools are placed on the UGP (OT_UGP_) and the RFM (OT_RFM_) allowing the measurement of their relative pose.

The PFMD was tested in distal femoral intra-articular fractures with a robotic system that consists of two robotic fracture manipulators (RFM) (Dagnino et al., 2017a) designed and manufactured in the Bristol Robotics Laboratory being able to apply manipulation forces and torques of 350 N/12 Nm. These fractures usually consist of two large fragments (i.e., type 33-C1, AO classification) and require manipulation of different size bone fragments. The PFMD connects the two RFMs with the bone fragments. Because of the potential trauma to the fragments, the device is attached in the mono-cortical fashion. The device consists of the Unique Geometry Pin (UGP) (Figure 1A), the Anchoring System (AS) (Figure 1B), and the Gripping System (GS) (seen in Figure 1C).

The UGP (Figure 1A) is a custom-designed non-cannulated orthopedic manipulation pin [6 mm diameter (D), 142 mm length (L)]. It has 4 distinctive cross-sections: *(i) gripping section* (L = 12 mm) a cylindrical section of 4 mm diameter, to be connected to the GS; *(ii) tool section* (L = 33 mm), a three-flat-faces unique geometry to which a tool (e.g., optical tool for real time tracking) can be mounted in a unique orientation, enabling the 3D imaging system (Dagnino et al., 2016a,b); *(iii) anchoring system section* (L = 67 mm), a two-flat-faces geometry to which the AS is fixed preventing rotation around the UGP axis; *(iv) threaded section* (L = 30 mm), an M6 metric thread (max. diam. 5.91 mm, min. diam. 4.74 mm, pitch 1 mm), screwed into a single cortical plane of the fragment. The specified pitch, diameter and number of turns of the thread were selected optimizing manufacturing limitations and pull-out characteristics (Chapman et al., 1996). Deformations of the UGP via the tool section are recorded using a commercial optical tracking system (Polaris Spectra, NDI Inc., tracking accuracy 0.25 mm).

The AS (Figure 1B_1_) is a custom designed system that firmly connects the UGP with a bone fragment using a holding ring called a Drilling Template (DT) and three or four 2 mm (diameter) stainless steel K-wires. The DT (Figure 1B_2_) has a total of 5 openings, one central with two-flat-faces for the UGP, and four circular ones for the K-wires. The surgeon drills the UGP into the bone fragment following the normal procedure for pin placement, then slides the DT into the *anchoring system section* and drills the 4 K-wires into the bone fragment through the holes on the DT. The K-wires cross through the holes of the DT and into the bone firmly stabilizing the UGP to the fragment.

The GS (Figure 1C) is mounted on the RFM end-effector and consists of an adjustable spherical joint that can freely orient a specially designed insert which fits in the *gripping section* of the UGP. This configuration ensures that the force/torque applied by the RFM is fully transferred to the bone fragment to achieve the desired anatomical reduction.

For the manual reduction trials, a specially instrumented handle was used to allow the surgeon to grasp the PFMD. The handle is instrumented with a 6DOF load-cell (FTSensor, IIT, Italy) to record forces and torques used in the manipulation maneuvers. A special cannulated version of the UGP (CV-UGP) is used for the electromagnetic tracking evaluation. This version is required to allow the placement of an electromagnetic sensor in a location that can provide information comparable to the optical tools described above.

### Simulation Using Finite Element Analysis

The main aim of the finite element analysis (FEA) was to assess structural properties, breaking points, and maximum theoretical deformations of the UGP and AS under typical forces applied in fracture surgeries. The software package used was Autodesk^Ⓡ^ Simulation Mechanical 2017.

The appropriate loading values used for this analysis have been established through discussions with orthopedic surgeons, analysis of various fracture cases (Dagnino et al., 2015), and *in-vivo* measured forces applied by the surgeons during lower limb surgical procedures (Georgilas et al., 2015). To this effect, the analysis has been simulated with the following loading scenarios, (a) torsional load for K-wire failure pattern (*T*_fail_ = 2.6 Nm), (b) average combined load (*F*_ave_ = 75.7 N, *T*_ave_ = 3.5 N), and (c) maximum combined load (*F*_max_ = 147 N, *T*_max_ = 6.8 Nm). Scenarios (b) and (c) are also conducted with the CV-UGP to ensure that this version performed similarly to the standard version. For the first scenario, the metric reported is the safety factor calculation for K-wires (i.e., probability of mechanical failure) as well as the deformation of the k-wires at the point of interface with the DT. For the latter scenarios, the metrics are the deformation of the *gripping section* (i.e., due to actuation).

#### Artificial Materials Laboratory Testing

**Figure 2:**
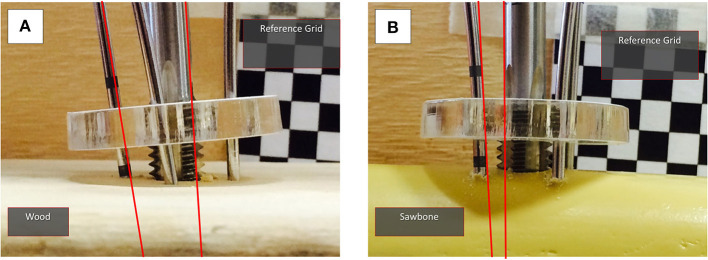
Laboratory testing experiments with **(A)** wood and **(B)** Sawbone model. The reference grid for the measurements can be seen and the angle between the UGP and one of the K-wires is indicated. The measurements were conducted in a series of still images with progressively increased torsional load, up to 2.6 Nm.

Following the FEA simulation experiments of the manipulation device, preliminary laboratory experiments on bone phantoms (Sawbones) and wood were conducted. The aim of the testing was to evaluate performance of the proposed device and, more specifically, to analyze how loading the K-wires affects stability of the device- bone attachment. During this test, a first prototype of the attachment device was drilled to a flat wood surface (Figure 2A) and a Sawbone femur model (Figure 2B). Four K-wires were inserted via the DT into the respective surface, and the UGP was rotated around the axis of the device with a progressively increased torsional load of up to 2.6 Nm. For these loading tests the metric reported is the calculated bending displacement of the K-wires at the point of interface with the DT defined as the angle between the UGP and the K-wires (red lines in Figure 2).

### Electromagnetic Tracking Evaluation

As described above, for measuring the position and deformation of the PFMD, an optical tracking method was used. In order to evaluate the measurements from these tools, an experiment to compare the displacement values with a different sensing modality, namely a commercial electromagnetic tracking tool (Aurora Tabletop Fieldig−6 DOF sensors, NDI Inc., tracking accuracy 0.8 mm), was performed. The PFMD with sensors for both tracking systems was placed into a swine leg sample (a food-chain trotter) and force and torque loading similar to the deformation tests described below was applied.

**Figure 3:**
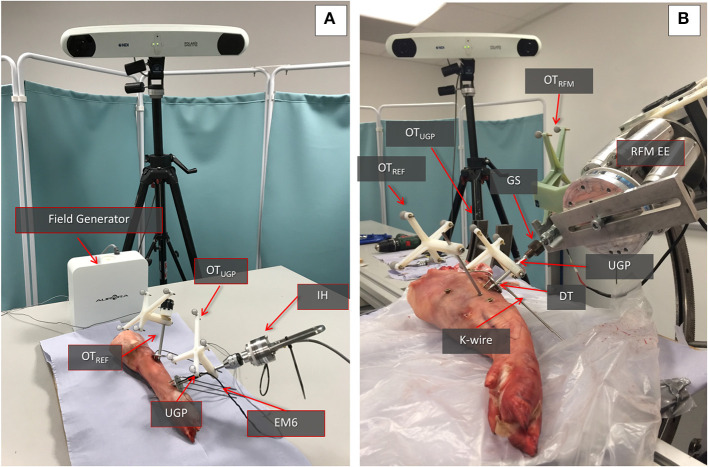
Swine trotter testing setup. **(A)** The elements of the electromagnetic tracking evaluation can be seen. The Instrumented handle (IH) and the Unique Geometry Pin (UGP) can be seen as well as the placement of the optical tools OT_UGP_ and OT_REF_ and the electromagnetic 6DOF sensor (EM6). **(B)** The key parts of the Robot-Bone Fixation System can be seen (GS, UGP, DT, K-wires), as well as the optical tools for the RFM (OT_RFM_), the UGP (OT_UGP_), and the optical tool used as reference for the calculations (OT_REF_).

The sample was freely placed on the table, since no absolute values were recorded, and the manipulation was performed using an instrumented handle (IH) to securely grasp the PFMD and apply external forces (Figure 3A). The IH had a load cell attached to measure forces applied, similar to the setup presented in Georgilas et al. (2015). Moreover, the table top field-generator of the Aurora system was placed in an optimal position to minimize following interference from ferromagnetic materials following the recommendation from Yaniv et al. (2009). The electromagnetic 6 DOF sensor (EM6) of the Aurora system is a 2.5 mm diameter coil-in-cable placed inside the PFMD with their major axis aligned. The metrics recorded were relative errors between the optical tool OT_UGP_ and the electromagnetic EM6. The first is referenced with respect to the OT_REF_ and the latter with respect to the field-generator. More details about the electromagnetic Tracking Evaluation can be found in Martins et al. (2018).

### *Ex-vivo* Animal Testing

The performance of the manipulation device has been evaluated in a second set of *ex-vivo* tests with a swine leg sample (a food-chain trotter, Figure 3B). The aim was to evaluate if the deformation of the device will follow the results collected in the simulation and the preliminary wood/Sawbone tests. The device was inserted into the trotter bone and the RFM applied different loads to assess deformations.

The sample is stabilized on the operating table using screws on a sacrificial material to emulate the use of a Taylor Spatial Frames (TSF) ring and Schanz pins in the clinical process. The UGP is drilled and tapped in the femur and secured using the DT and K-wires. Three optical tools with markers were used to track the relative motion of the UGP. The OT_REF_ is the reference frame in respect to which all translations and rotations are calculated. It is fixed to the trotter. The OT_UGP_ provides information for the *tool section* and is used for the reduction (i.e., evaluates the bending of the UGP). The OT_RFM_ provides pose of the end-effector of the RFM and the GS and is used to guide the robot (i.e., evaluates the error between a desired and actual positioning of the bone). The metrics chosen for this testing is the relevant displacement between the *gripping section* and the UGP as measured by the relative motion between OT_RFM_ and OT_UGP_.

### Cadaveric Trial

The device was finally evaluated through reductions of complete articular distal femur fractures on 7 human cadaveric specimens. The specimens used were right and left male (*n* = 4) and female (*n* = 3) lower limbs with no bone defects on which the desired fractures were initially created. The cadaveric specimens were kept in conditions of −18°C. During handling of the specimens and collection of data, all national requirements and guidelines for ethical use of human tissue were followed.

**Figure 4:**
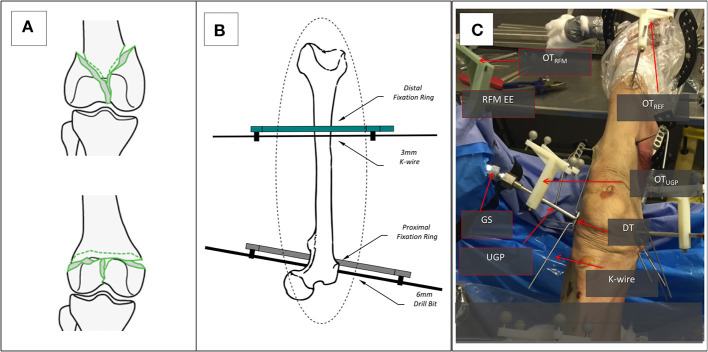
The setup for the cadaveric study. **(A)** The two types of generated 33-C1 fractures Y-shape and T-shape. **(B)** The stabilization of the femur with the Proximal and Distal Rings and the use of a 6 mm Long Drill Bit and a 3 mm K-wire. **(C)** The entire setup for one of the RFM, the UGP, and GS can be seen attached to the RFM end-effector (EE). The UGP is secured to the fragment with the use of the DT and a number of K-wires. The optical tools are also visible, the OT_RFM_, the OT_UGP_, and the OT_REF_ on the shaft of the femur proximal to the hip.

Given the nature of the measurements (i.e., application of forces and comparison of movement) there are two important phases to the preparation of the specimens for the study, the creation of the fractures and the stabilization to the operating table. For the creation of appropriate fracture shapes [T and Y, 33-C1 (AO Foundation, 2015), Figure 4A] in a predictable and reproducible manner, an accepted technique of osteotomy was used. The stabilization of the specimens was done following existing clinical procedures and special effort was made to ensure that the process was emulating *in-vivo* conditions given the lack of a hip joint. For the purposes of the study, two points of stabilization were used, the first through the femoral head and the second more distantly in the shaft, closer to the fracture. In the femoral head, a long drill bit of 6 mm diameter was used to secure the bone, while in the distal point, a 3 mm k-wire was used in an angle to the drill bit as seen from the axis of the limb. Both points were secured on the TSF rings, a typical orthopedic approach for percutaneous stabilization of bones. With real patients, only the distal point would be utilized since the body weight via the hip joint would provide enough proximal stability (Figure 4B). The rest of the setup for the manipulation device was similar to the *ex-vivo* animal study with the same number of optical tools for the UGP position, Figure 4C. The metrics chosen for this testing is the relevant displacement between the *gripping section* and the UGP as measured from the relative motion between OT_RFM_ and OT_UGP_.

**Figure 5:**
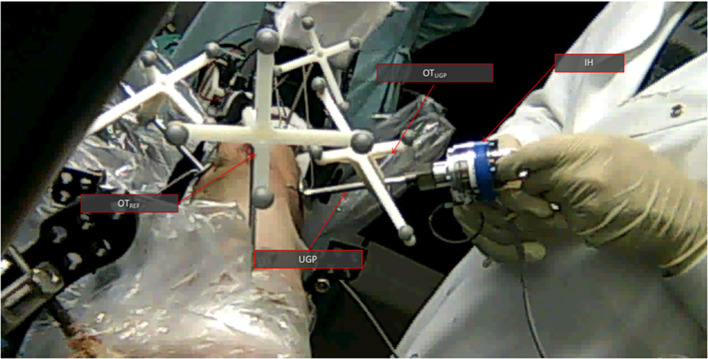
The manual cadaveric trial setup. View from the proximal side of the femur toward the distal. The UGP is visible as well as the instrumented handle (IH) The optical tools are also visible OT_UGP_ for the tracking of the PFMD and OT_REF_ as a reference. Feedback from the former is helping with the reduction.

In addition to the robot-assisted testing, manual reduction maneuvers were performed on four cadaveric specimens (#1, #2, #3, #6). All cadaver specimens had similar fracture patterns. The specimens reported here where the first to be manually reduced and was found that performing manual manipulation in all specimens would not significantly impact the obtained results. The surgeon used the instrumented handle (IH) to securely grasp the PFMD and reduce the fragments (Figure 5). The reduction was guided using the optical tool feedback (motion of OT_UGP_ in respect to OT_REF_). The final results were assessed using intra-operative fluoroscopy.

In the results and discussion sections, all simulated values are given as absolute values, given the deterministic nature of the process. For the experimental data, individual values are given to the second decimal, while all averaged experimental data will be presented along with the standard deviation (SD) of the sample and the confidence interval (CI) at 50%. For the simulation data, the forces reported are absolute while for the cadaveric and *ex-vivo* swine trials, the forces will be given as a range, since stable (<10% fluctuation) force application was practically difficult to implement, especially for the manual reductions.

## Results and Discussion

### Finite Element Analysis Results

In the FEA simulated K-wire testing scenario (*T*_fail_ = 2.6 Nm), the yield stress safety factor between 0.8 and 2.5 was calculated in the interface with the bone surface. This indicates that the K-wires undergo plastic deformation but do not destructively break. Moreover, the simulation revealed that at the point of interface, the K-wires were deformed by 0.16 mm. Similar observation was made during the experimental process (Sawbones model, wood) and cadaveric tests where the K-wires bent on the interface with the bone. Also, at the laboratory testing the displacement at the interface point of K-wire and DT was measured at 0.23 mm, SD 0.03 mm, CI_50%_ 0.02 mm.

### Electromagnetic Evaluation Results

The experiments using the electromagnetic tracking demonstrated that the optical tracking can yield comparable results. Specifically, the experiments with the *ex-vivo* swine for the Aurora and Polaris systems gave an average error between the two of 0.5 mm, SD 0.29 mm, CI_50%_ 0.01 mm. This is in-line with the accuracy of the two systems which is 0.8 and 0.3 mm RMS for Aurora and Polaris, respectively. For the rest of the result section the values presented are measurements collected with the Polaris system.

### Deformation Experiments

Regarding the deformation experiments, the simulation for the *gripping section* suggested for loading *F*_ave_ = 75.7 N/*T*_ave_ = 3.5 Nm a deformation of 2.8 mm and for loading *F*_max_ = 147 N/*T*_max_ = 6.8 Nm a deformation of 5.8 mm. For all the physical experiments (*ex-vivo* swine and cadavers) the respective average values are 3.03 mm, SD 0.64 mm, CI_50%_ 0.16 mm and 5.19 mm, SD 0.46 mm, CI_50%_ 0.18 mm for similar force ranges. Table 1 summarizes the data collected for all the experiments, both *ex-vivo* swine and cadaveric, with average values for the respective specimen types. For the manual reductions, the deformation of the PFMD was not measured because the optical tool was not attached to the handle to provide relative motion data. The force applied by the surgeon and measured from the IH was on average 65 N, SD 8.66 N, CI_50%_ 2.2 N.

**Table 1:** Displacement data for all experimental results.

**Loading**	**Metric**	**Swine1**	**Swine2**	**Avg**.	**Cadaveric specimen**							**Avg**.
					**#1**	**#2**	**#3**	**#4**	**#5**	**#6**	**#7**	
80 N ± 8 N	RMD	2.79	3.14	2.97	2.60	4.03	3.82	rej.	2.82	2.04	rej.	3.06
		1.48	1.85		1.12	1.14	1.48	rej.	0.66	0.26	rej.	
150 ± 15 N	RMD	nd.	4.44		nd.	nd.	nd.	5.55	nd.	nd.	4.83	5.19
		nd.	2.71		nd.	nd.	nd.	2.75	nd.	nd.	1.85	

*RMD, resultant maximum displacement (translational and rotational); nd., no data collected due to lack of necessary loading for reduction; rej., data rejected due to issues in the data collection process*.

### Reduction Quality

Regarding the quality of the reduction, all the specimens for the robot-assisted trials were reduced in acceptable or borderline manner with an average translational difference of 1.17 mm, SD 0.56 mm, CI_50%_ 0.14 mm as reported in Dagnino et al. (2017a); where acceptable means that the reduction accuracy was ≈1 mm, ≈5° and borderline that the accuracy was higher but the fracture is clinically considered reduced. For the manual reductions, the four specimens have been reduced to acceptable levels as confirmed by post-operation fluoroscopy and are reported in Table 2 with an average translational difference of 1.24 mm, SD 0.44 mm, CI_50%_ 0.11 mm.

**Table 2:** Reduction accuracy of manual maneuvers—cadaveric speciments.

**Metric**	**#1**	**#2**	**#3**	**#6**
Root-mean-squared-error (RMSE)	1.35 ± 0.34 mm	0.94 ± 0.75 mm	0.91 ± 0.38 mm	1.46 ± 0.58 mm
	0.32 ± 0.19°	4.55 ± 1.93°	5.19 ± 1.55°	5.65 ± 1.4°

### Discussion of Results

The FEA simulation data predicted that there will be a plastic deformation of the K-wires (safety factor between 0.8 and 2.5) and this has been qualitatively confirmed during the experimental (Sawbones models, wood) and cadaveric tests. A further finding to strengthen this point was also the artificial material laboratory testing with the 0.23 mm displacement which caused the K-wires to plastically deform but not break in any sample. As a secondary effect of the plastic deformation that happens in place, the interface between the DT and the K-wire is strengthened since the latter is tensioning, increasing the rotational stability of the PFMD. Regarding the main pin deformation, the close similarity between the simulation, *ex-vivo* laboratory, and cadaveric data, with measurements at 80 N ± 8 N of 2.8, 2.97, and 3.06 mm, and at 150 ± 15 N of 5.8, 4.44, and 5.19 mm respectively, demonstrate that the UGP can be well-described by the simulation model. This can enable the use of the UGP position to evaluate reduction accuracy and also act as a guidance system both for manual and for robot-assisted fracture manipulations.

It has also been demonstrated that the reduction accuracy for the cadaveric specimens could be of acceptable quality for clinical practice. This has been reported in Dagnino et al. (2017a) and summarized here as an average translation accuracy of 1.17 mm, SD 0.56 mm, CI_50%_ 0.14 mm. The accuracy is achieved despite the above deformation since the robot-assisted system can compensate via its given reduction accuracy of 1.15 mm 1.3° d, reported in Dagnino et al. (2016a) and registration accuracy of 1.15 mm ± 0.8 mm reported in Dagnino et al. (2017b). Similar levels of reduction accuracy have been recorded with the manual manipulation using the proposed PFMD. The reductions achieved have an average translational accuracy of 1.24 mm, SD 0.44 mm, CI_50%_ 0.11 mm. The forces reported were of similar range but it must be noted that the manual reductions required less force because the other fragments were not constrained.

When comparing the performance and capabilities of the proposed PFMD with existing devices for long-bone manipulation we can observe that the proposed device performs better or similarly. When compared to external fixation configurations like the one presented in Gösling et al. (2006) the maximum forces and torques applied are lower, 165 N and 6.8 Nm vs. 411 N and 74 Nm, but in this study we report good reduction of the articular surface vs. acceptable reduction of the shaft for Gösling et al. (no reduction quality is provided). Similar is the comparison with the device in Füchtmeier et al. (2004) where the applicable forces have a maximum of 250 N (no reduction quality is provided). Compared to Ye et al. (2012), the results are in the same range of accuracies. In that work the reduction accuracy of the proposed method has an average positional error of 1.03 mm, SD 0.59 mm but this is based on the mechanical analysis of the device and not under realistic conditions, e.g., effects of soft tissue, and full loading. When compared with locking plates and screws configurations similar to Schmucki et al. (2004) for equivalent loadings of 165 N the differences are significant with distortions of up to 4 mm. The performance is comparable for lower forces of up to 75 N. Finally, when compared with the *Three-Point-Device* from Weber-Spickschen et al. (2010) the proposed PFMD is similar to the 40° version with average relative translational movements for the latter of 1.18 mm, SD 0.44 mm. None the less the physical size and necessary bone anchoring surface of that device is significantly larger especially for the 90° version that can achieve even lower average relative translational movements.

## Conclusions

This article describes a new proposed device for the percutaneous manipulation of bone. Although there are many studies that consider long bone manipulation, to the best of the authors' knowledge, no similar study has been conducted for the manipulation of intra-articular fragments. This type of fragment poses a challenging problem given its relatively small size and often deteriorated bone quality.

One of the key considerations regarding the proposed PFMD is that its smaller size does not compromise the reduction accuracy due to higher degrees of deformation under external loading. The current study demonstrated that there is not such an issue. The simulated, laboratory, *ex-vivo* swine and cadaveric trials confirmed this point with reduction accuracies that exhibited acceptable clinical levels. Moreover, the results demonstrate that the proposed PFMD is a good option for intra-articular fracture reduction when compared to the capabilities of existing methods for long bones reduction and taking into account the different nature of the clinical conditions and requirements. Namely the system performs under lower loads but it can retain a high degree of accuracy, similar to values reported for these systems.

The percutaneous manipulation and reduction of a fracture is preferable to open reduction techniques as it avoids the morbidity associated with larger wounds and stripping of the soft tissue envelope in intra-articular fractures (McCann et al., 2011). However, the inability to reliably bring about and maintain reduction so that fixation can be achieved has meant that clinicians have avoided this technique for most areas of fracture surgery, in particular that involving larger fragments. The proposed system offers a solution which may change clinical practice.

In conclusion, the study demonstrated that the proposed PFMD is suitable for the manipulation of small, odd-shaped, intra-articular fragments. With the use of an appropriate compensation method the deformation does not have a detrimental effect to the reduction accuracy. The next steps will be to test this device in a larger number of specimens and implement the compensation method.

## Data Availability Statement

The datasets generated for this study are available on request to the corresponding author.

## Ethics Statement

This study was carried out in accordance with the recommendations of National Research Ethics Committee, REC. The protocol was approved by the National Research Ethics Committee, REC with reference: 15/WM/0038, UK.

## Author Contributions

IG, GD, and SD contributed conception and design of the study. PK designed the original version of the PFMD. KG performed the FEA of the PFMD. BA designed and performed the electromagnetic part of the study. SM collected the data from the Artificial Materials Laboratory Testing. PT and RA performed the cadaveric part of the study. IG wrote the draft of the manuscript. IG, GD, BA, and SD wrote sections of the manuscript. All authors contributed to manuscript revision, read and approved the submitted version.

### Conflict of Interest

The authors declare that the research was conducted in the absence of any commercial or financial relationships that could be construed as a potential conflict of interest.
